# Life Under Hypoxia Lowers Blood Glucose Independently of Effects on Appetite and Body Weight in Mice

**DOI:** 10.3389/fendo.2018.00490

**Published:** 2018-08-28

**Authors:** Sameer Abu Eid, Martina T. Hackl, Mairam Kaplanian, Max-Paul Winter, Doris Kaltenecker, Richard Moriggl, Anton Luger, Thomas Scherer, Clemens Fürnsinn

**Affiliations:** ^1^Division of Endocrinology & Metabolism, Department of Medicine III, Medical University of Vienna, Vienna, Austria; ^2^Division of Cardiology, Department of Medicine II, Medical University of Vienna, Vienna, Austria; ^3^Institute of Animal Breeding and Genetics, University of Veterinary Medicine Vienna, Vienna, Austria; ^4^Ludwig Boltzmann Institute for Cancer Research, Vienna, Austria; ^5^Medical University of Vienna, Vienna, Austria

**Keywords:** hypoxia, glucose, appetite, body weight, insulin sensitivity

## Abstract

Blood glucose and the prevalence of diabetes are lower in mountain than lowland dwellers, which could among other factors be due to reduced oxygen availability. To investigate metabolic adaptations to life under hypoxia, male mice on high fat diet (HFD) were continuously maintained at 10% O_2_. At variance to preceding studies, the protocol was designed to dissect direct metabolic effects from such mediated indirectly via hypoxia-induced reductions in appetite and weight gain. This was achieved by two separate control groups on normal air, one with free access to HFD, and one fed restrictedly in order to obtain a weight curve matching that of hypoxia-exposed mice. Comparable body weight in restrictedly fed and hypoxic mice was achieved by similar reductions in calorie intake (−22%) and was associated with parallel effects on body composition as well as on circulating insulin, leptin, FGF-21, and adiponectin. Whereas the effects of hypoxia on the above parameters could thus be attributed entirely to blunted weight gain, hypoxia improved glucose homeostasis in part independently of body weight (fasted blood glucose, mmol/l: freely fed control, 10.2 ± 0.7; weight-matched control, 8.0 ± 0.3; hypoxia, 6.8 ± 0.2; *p* < 0.007 each; AUC in the glucose tolerance test, mol/l^*^min: freely fed control, 2.54 ± 0.15; weight-matched control, 1.86 ± 0.08; hypoxia, 1.67 ± 0.05; *p* < 0.05 each). Although counterintuitive to lowering of glycemia, insulin sensitivity appeared to be impaired in animals adapted to hypoxia: In the insulin tolerance test, hypoxia-treated mice started off with lower glycaemia than their weight-matched controls (initial blood glucose, mmol/l: freely fed control, 11.5 ± 0.7; weight-matched control, 9.4 ± 0.3; hypoxia, 8.1 ± 0.2; *p* < 0.02 each), but showed a weaker response to insulin (final blood glucose, mmol/l: freely fed control, 7.0 ± 0.3; weight-matched control, 4.5 ± 0.2; hypoxia, 5.5 ± 0.3; *p* < 0.01 each). Furthermore, hypoxia weight-independently reduced hepatic steatosis as normalized to total body fat, suggesting a shift in the relative distribution of triglycerides from liver to fat (mg/g liver triglycerides per g total fat mass: freely fed control, 10.3 ± 0.6; weight-matched control, 5.6 ± 0.3; hypoxia, 4.0 ± 0.2; *p* < 0.0004 each). The results show that exposure of HFD-fed mice to continuous hypoxia leads to a unique metabolic phenotype characterized by improved glucose homeostasis along with evidence for impaired rather than enhanced insulin sensitivity.

## Introduction

Basal blood glucose and insulin resistance, as well as the prevalence of obesity and diabetes have been shown to be lower in mountain than lowland dwellers, which among many other factors could be due to the lower partial oxygen pressure at high altitude (1–5). A multitude of experimental studies investigated the metabolic consequences of hypoxia and brought forward very diverse findings, which could relate to the variety of different protocols applied. Factors that may affect the outcome include, e.g., differences in the extent, temporal pattern, and duration of oxygen deficiency, as well as the nutritional state and sample collection under concurrent hypoxia vs. after return to normal air.

Some rodent experiments were designed to mimic the episodes of recurrent short-term oxygen deficiency as typically occurring in obstructive sleep apnea. The majority of such studies, in which rodents were exposed to rapidly alternating cycles of hypoxia and normoxia, found detrimental effects on glucose tolerance, insulin sensitivity and hepatic steatosis (6–10). Nevertheless, divergent findings of lower blood glucose in response to treatment with brief bouts or prolonged periods of intermittent hypoxia have also been reported (11–14). With regard to a stay at high altitude or continuous exposure to experimental hypoxia, short periods for less than a week (as may be associated with mountain sickness) have likewise been associated predominantly with a deterioration of glucose homeostasis (3, 15–18). But opposing outcomes exist also for this type of protocol (19). While the metabolic derangements seen with intermittent hypoxia or with short term continuous hypoxia could in part be due to a stress response associated with adrenergic signaling and/or a rise in plasma corticosteroids (14, 16, 17, 19–22), the majority of studies in humans and animals suggested a turn toward normal or even improved glucose homeostasis with prolonged life under continuous low oxygen availability (3, 11, 13, 16, 18, 19, 23, 24).

The evidence for an anti-hyperglycemic potential of prolonged life under hypoxia, however, is presently hampered, firstly, by occasional use of glucose meters, which are known to underreport the blood glucose concentration in the presence of a high hematocrit, and secondly, by an unclear contribution of indirect effects via changes in food consumption. Although the anorectic action of mountain ascents and experimental hypoxia is undisputed (12, 19, 25–27), no study in humans or animals has yet convincingly dissected, if or to what extent metabolic benefits arising from a hypoxic environment reach beyond the trivial consequences of a spoilt appetite that could be accompanied by the well-known metabolic attributes of leanness.

Against this background, the present study set out to describe the metabolic phenotype of mice subject to prolonged maintenance under continuous hypoxia, while simultaneously correcting for changes in food intake and body weight. At variance with many preceding mouse studies targeting obstructive sleep apnea, our protocol is designed to analyze lasting metabolic adaptations to continuous hypoxia such as highland dwellers, mountain climbers or trekking tourists will undergo.

## Materials and methods

### Animals

Forty male C57BL/6J mice were purchased from Charles River Laboratories (Sulzfeld, Germany) at an age of 6 weeks. They were housed in groups of four in polycarbonate cages provided with wood-based bedding (Hygienic Animal Bedding, J. Rettenmaier & Söhne, Rosenberg, Germany) and had free access to drinking water. Constant room temperature and an artificial 12 h dark/12 h light cycle were always provided.

Until an age of 10 weeks, mice had free access to conventional rodent chow diet (sniff R/M-H, sniff Spezialdiäten GmbH; Soest, Germany). Subsequently, they were allocated to 4 groups matched for body weight, body composition, and basal blood glucose. The groups were maintained under the following conditions for 3 months: The two groups most important for answering our primary question about the weight-neutral effects of life under hypoxia were (i) mice with free access to high fat diet (HFD; 60% of calories as fat; Cat.# D12492, Research Diets Inc., New Brunswick, NJ, USA) while maintained under hypoxia (*n* = 12); and (ii) mice with restricted access to HFD (one portion per day), aiming at a weight curve that matched the curve of their hypoxia exposed counterparts (*n* = 12). Two additional groups were studied to pinpoint the roles of the composition and the amount of food consumed: (iii) mice with free access to conventional chow diet (*n* = 8); and (iv) mice with free access to HFD. Note that HFD feeding and exposure to hypoxia were started at the same time and, hence, our protocol was designed to study hypoxia dependent prevention, not reversal, of HFD induced metabolic deterioration.

Exposure to hypoxia was conducted under normobaric conditions in a chamber equipped with an oxygen sensor. The system allowed continuous maintenance of the target oxygen level of 10% by diluting the compartment air with an appropriate amount of nitrogen (BioSpherix, Lacona, USA). The chamber was briefly opened once daily to provide clean cages and to avoid accumulation of humidity and smell.

The study was in line with effective national and international guidelines and law, and all procedures followed the principles of good laboratory animal care. The protocol was approved by the Austrian Federal Ministry of Science, Research and Economy.

### Protocols and procedures

#### Time schedule

**Figure 1:**
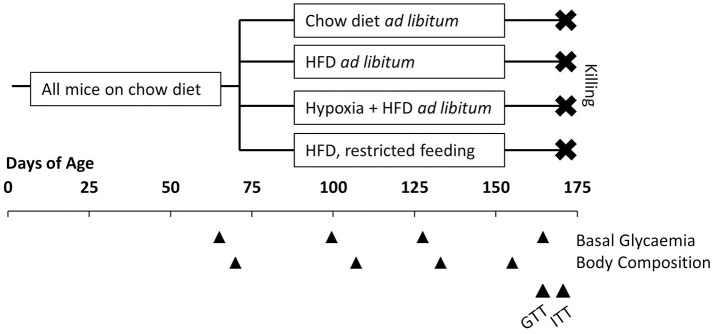
Illustration of the experimental design. All mice had free access to conventional chow diet before allocated to four groups at the age of 10 weeks: free access to chow diet (*n* = 8); free access to high fat diet (HFD; *n* = 8); free access to HFD under hypoxia (*n* = 12); restricted feeding of HFD so to obtain a weight curve as in hypoxia-exposed group (*n* = 12). Basal glycaemia and body composition were measured at intervals of ~1 month. In the last 2 weeks before killing a glucose tolerance test (GTT) and an insulin tolerance test (ITT) were performed.

Body weight and food intake were determined and documented at least twice weekly. Before and ~1, 2, and 3 months after dividing the mice into groups, basal blood glucose and body composition (lean and fat mass; EchoMRI, Houston, TX, USA) were measured. During the last 2 weeks of the study, a glucose tolerance test (GTT) and an insulin tolerance test (ITT) were performed. Approximately 3 months after starting the investigations, mice were removed from hypoxia and fasted for 4 h, before they were deeply anesthetized by inhalation of isoflurane and killed by bleeding via heart puncture. Samples of plasma and the liver were rapidly collected and stored at −80°C for later analysis. The experimental design is graphically illustrated in Figure 1.

It is important to note that mice were removed from hypoxia and kept under normal air for 24 h, before any measurement of basal glycemia, a GTT, or an ITT were performed. They were on normal air for 4 h before the terminal sampling of plasma and tissues. Hence, our study was designed to describe lasting metabolic adaptations to hypoxia, but not to record acute and rapidly reversible effects of oxygen deficiency.

#### Measurement of blood glucose

Blood glucose was measured in duplicate in capillary blood collected from the tip of the tail with a hand-held glucose meter device (OneTouch Ultra, LifeScan Inc., Milpitas, CA, USA). Using another group of obese mice exposed to the applied hypoxia regimen, we carefully examined the hematocrit sensitivity of this device. A rise in the hematocrit found in that mice (hypoxia, 54.2 ± 0.9%, vs. controls, 44.7 ± 1.2%; *p* < 0.0001) confirmed the effectivity of the employed hypoxia treatment, but a hematocrit increase of this magnitude has been described by others to cause a 10% underestimation of blood glucose by the employed glucose meter (28, 29). After confirming this 10% deviation in house with five hematocrit independent methods for the measurement of glycemia, a correction factor of 1.1 was applied to all glucose values from hypoxia-exposed animals.

#### GTT and ITT

The GTT and the ITT were performed along protocols employed earlier (30, 31). In the GTT, blood glucose was measured in mice fasted for 8 h and immediately thereafter, glucose solution was injected intraperitoneally (1.5 g/kg; 4.5 ml/kg). The resulting excursion of blood glucose was documented 20, 40, 60, 90, and 120 min after glucose administration. For the ITT, food was withdrawn 4 h before an intraperitoneal injection of human insulin (NovoRapid from Novo Nordisk, Bagsvaerd, Denmark; 0.75 U/kg; 5 ml/kg) with measurements of blood glucose immediately before (0 min) and 15, 30, 45, and 60 min after the injection. In mice showing a blood glucose value <2 mmol/l at any time point, the ITT was immediately terminated by an injection of glucose solution. Due to the large number of mice, the tests were performed in two runs on two subsequent days, with 4 mice from each group tested on the second day.

#### Sample analysis

Hormone concentrations in plasma sampled at the time of killing were measured with the following commercially available kits: insulin, Ultrasensitive Mouse Insulin ELISA (Mercodia, Uppsala, Sweden); leptin, Metabolism & Endocrinology Multiplex Assays (Luminex, Merck Millipore, Burlington, MA, USA); adiponectin, Quantikine ELISA Mouse Adiponectin/Acrp30 (R&D Systems, MN, USA); fibroblast growth factor-21 (FGF-21), Quantikine ELISA Mouse/Rat FGF-21 (R&D Systems); corticosterone, Corticosterone ELISA (Enzo Life Sciences, Farmingdale, NY, USA). Hepatic triglycerides were determined after extraction according to Folch (32) using the Triglyceride Determination Kit from Sigma Aldrich (St. Louis, MO, USA).

### Statistics

Results are given as means ± S.E.M. Pairwise group comparison was done by two-tailed Student's *t*-tests or by 2-way ANOVA for repeated measurements. Correlations were analyzed according to Pearson. A *p* < 0.05 was considered significant.

## Results

### Food intake, body weight, and body composition (Figure 2)

**Figure 2:**
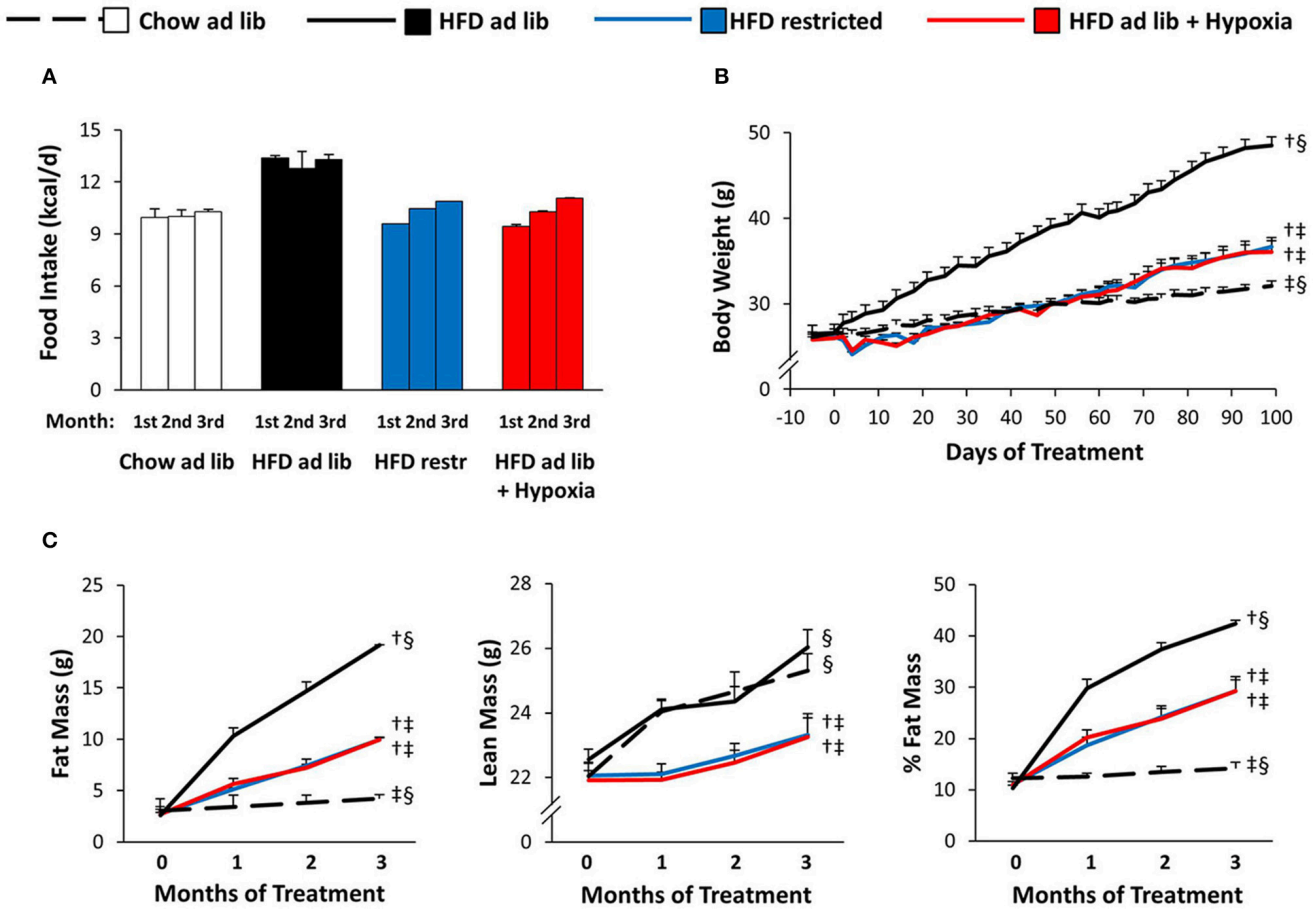
Food intake **(A)**, body weight **(B)**, and body composition **(C)** in four groups of mice maintained for 3 months under different conditions: free access to chow diet (Chow ad lib; *n* = 8); free access to high fat diet (HFD ad lib; *n* = 8); free access to HFD under hypoxia (HDF ad lib + Hypoxia; *n* = 12); restricted access to HFD, weight-matched with the hypoxia-exposed group (HFD restricted; *n* = 12). Means ± SEM. Food intake is reported descriptively without calculation of *p*-values, because it could be determined per cage only (*n* = 2 or 3 per group). For body weight and body composition: *p* < 0.05 by 2-way ANOVA for repeated measurements (**B**, effect of interaction time*group; **C**, effect of group): ^†^vs. Chow ad lib, ^‡^vs. HFD ad lib, ^§^vs. HFD restricted.

Hypoxia markedly reduced voluntary HFD consumption, which leveled off in the course of the study with reductions by approximately −30% during the first month and −20% during the last month (HFD *ad libitum* vs. HFD+hypoxia; Figure 2A). The amount of HFD, to which mice at normal air had to be restricted in order to obtain a similar weight curve as in those living under hypoxia, was almost the same as what the hypoxia exposed mice consumed deliberately (HFD restricted vs. HFD+hypoxia; Figures 2A,B). While the type of food provided *ad libitum* did not affect the gain in lean body mass (chow *ad libitum* vs. HFD *ad libitum*; Figure 2C), mice with free access to HFD consumed almost 30% more calories and gained approximately 14-fold more fat mass than those on chow diet (chow *ad libitum* vs. HFD *ad libitum*; Figures 2A,C). As compared to these two groups, gain of lean mass was blunted to a similar extent in hypoxic and restrictedly fed mice, implicating that the two interventions affected food efficiency and body composition in a similar manner (HFD restricted vs. HFD+hypoxia; Figure 2C).

### Circulating signaling molecules (Figure 3)

**Figure 3:**
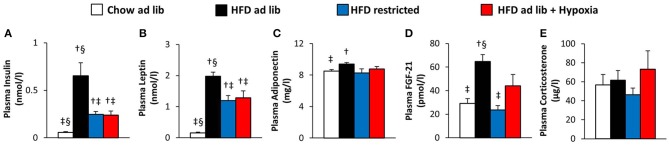
Plasma concentrations of insulin **(A)**, leptin **(B)**, adiponectin **(C)**, FGF-21 **(D)**, and corticosterone **(E)** in four groups of mice after 3 months under different conditions: free access to chow diet (Chow ad lib; *n* = 8); free access to high fat diet (HFD ad lib; *n* = 8); free access to HFD under hypoxia (HDF ad lib + Hypoxia; *n* = 12); restricted access to HFD, weight-matched with the hypoxia-exposed group (HFD restricted; *n* = 12). Means ± SEM; *p* < 0.05 by Student's *t*-test: ^†^vs. Chow ad lib, ^‡^vs. HFD ad lib, ^§^vs. HFD restricted.

Plasma concentrations of insulin and leptin increased markedly with HFD-induced obesity (chow *ad libitum* vs. HFD *ad libitum*; Figures 3A,B). These elevations were partly reversed in association with the reductions in food intake and fat mass observed in both, hypoxia-exposed and restrictedly fed mice (Figures 3A,B). Plasma adiponectin showed a minor increase in association with HFD induced obesity (chow *ad libitum* vs. HFD *ad libitum*; Figure 3C), which is at variance to most, but not all reports from the literature [e.g. (33–36)], and which was reversed with amelioration of obesity by hypoxia or restricted feeding (Figure 3C). Obesity distinctly increased plasma FGF-21 (chow *ad libitum* vs. HFD *ad libitum*; Figure 3D), which showed a trend toward higher concentrations under exposure to hypoxia than under food restriction (pmol/l: hypoxia, 44.1 ± 9.5, vs. weight matched control, 23.7 ± 3.6; *p* = 0.058; Figure 3D). Plasma corticosterone was not significantly different between the groups (Figure 3E).

### Blood glucose and glucose tolerance (Figure 4)

**Figure 4:**
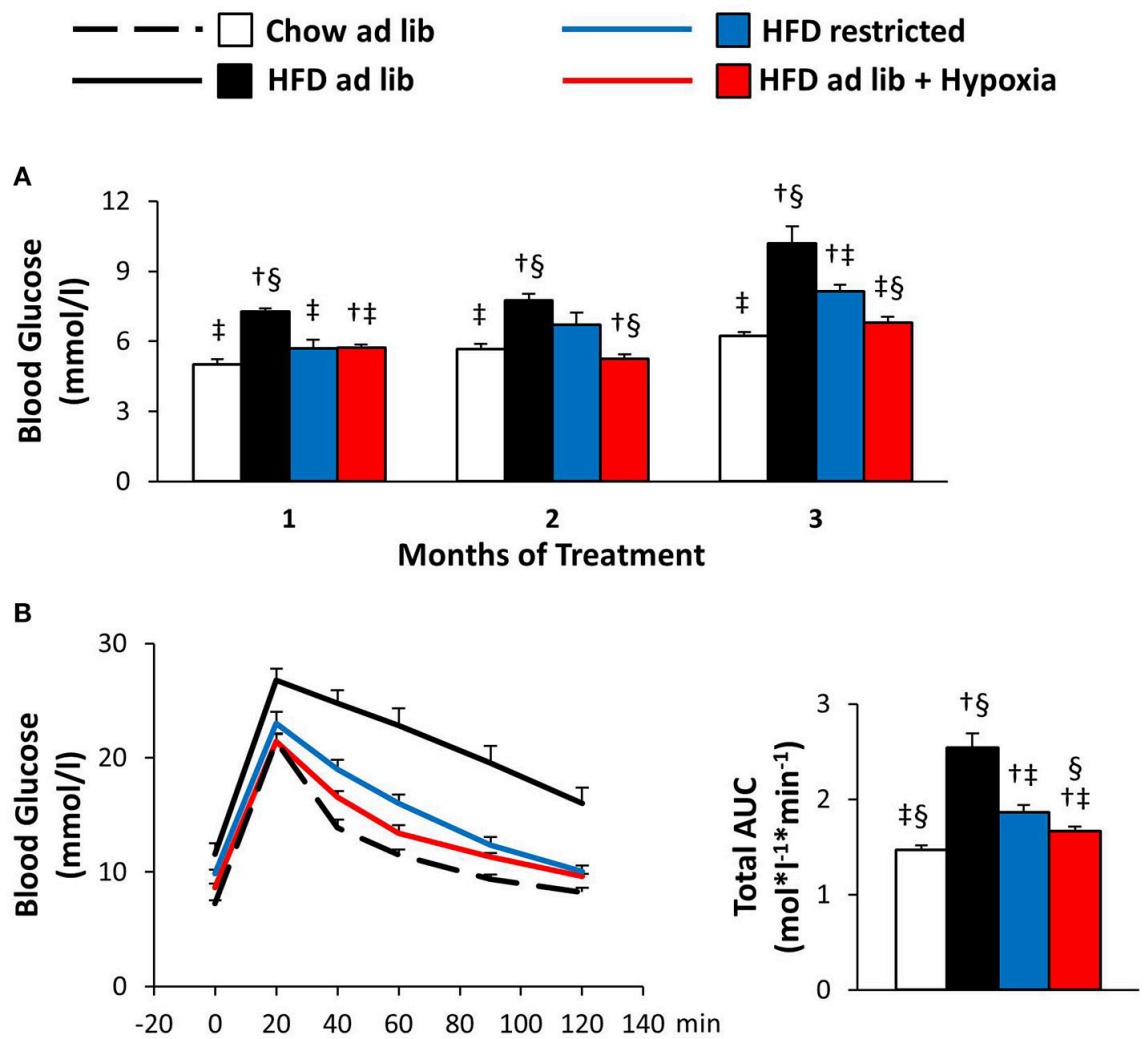
Basal blood glucose **(A)** and glucose tolerance test **(B)** in four groups of mice after 3 months under different conditions: free access to chow diet (Chow ad lib; *n* = 8); free access to high fat diet (HFD ad lib; *n* = 8); free access to HFD under hypoxia (HDF ad lib + Hypoxia; *n* = 12); restricted access to HFD, weight-matched with the hypoxia-exposed group (HFD restricted; *n* = 12). Means ± SEM; AUC, area under the glucose curve; *p* < 0.05 by Student's *t*-test: bar graphs, ^†^vs. Chow ad lib, ^‡^vs. HFD ad lib, ^§^vs. HFD restricted.

Obesity caused by free access to HFD caused marked deteriorations of basal blood glucose and glucose tolerance (chow *ad libitum* vs. HFD *ad libitum*; Figures 4A,B). Reduced consumption of HFD ameliorated hyperglycemia (HFD *ad libitum* vs. HFD restricted, and HFD *ad libitum* vs. HFD+hypoxia; Figures 4A,B) and, with some delay, maintenance in a hypoxic environment even normalized basal blood glucose to concentrations as circulating in chow fed lean mice (chow *ad libitum* vs. HFD+hypoxia; Figure 4A). In the GTT (Figure 4B), it is of note that the weight independent improvement of the total area under the glucose curve by hypoxia can be predominantly attributed to the lowering of basal glycemia, whereas the incremental glucose excursion was hardly affected (HFD restricted vs. HFD+hypoxia; Figure 4B; incremental area under the glucose curve: hypoxia, 0.63 ± 0.06, vs. weight-matched control, 0.68 ± 0.09 mol^*^l^−1*^min^−1^; ns).

### Insulin tolerance (Figure 5)

**Figure 5:**
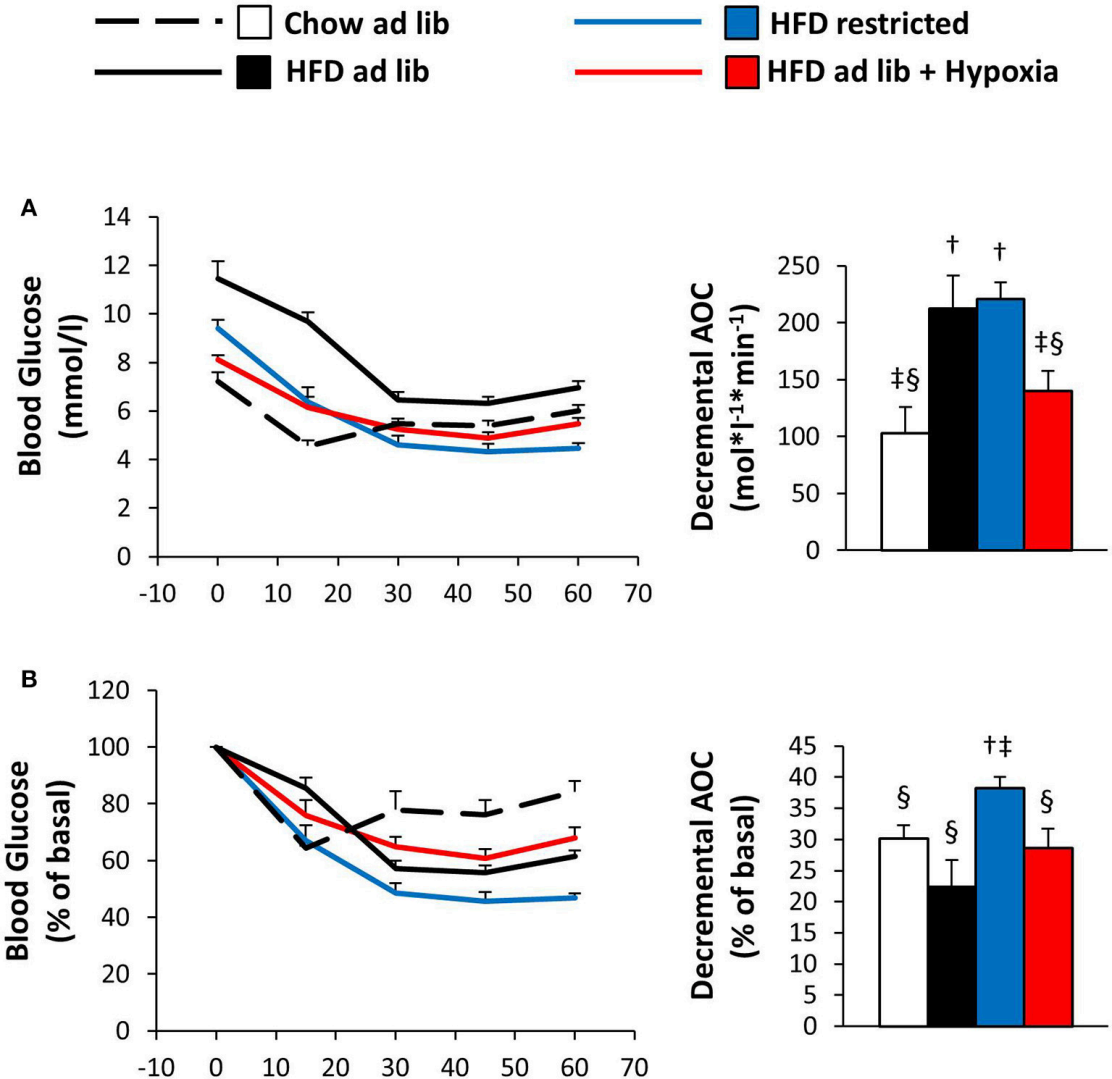
Blood glucose curves during the insulin tolerance test given as absolute concentrations **(A)** or as relative (%) changes vs. the starting value **(B)** in four groups of mice after 3 months under different conditions: free access to chow diet (Chow ad lib; *n* = 8); free access to high fat diet (HFD ad lib; *n* = 8); free access to HFD under hypoxia (HDF ad lib + Hypoxia; *n* = 12); restricted access to HFD, weight-matched with the hypoxia-exposed group (HFD restricted; *n* = 12). Means ± SEM; AOC, area over the glucose curve; *p* < 0.05 by Student's *t*-test: bar graphs, ^†^vs. Chow ad lib, ^‡^vs. HFD ad lib, ^§^vs. HFD restricted.

The ITT results in Figure 5 are depicted both in absolute values and in % change from the starting value. In line with obesity associated insulin resistance, the initial fall in blood glucose was steeper in mice with free access to chow vs. HFD. During the later course of the ITT, when counter regulation is known to be of major influence (37), blood glucose rose in the chow fed lean mice, whereas it continued to fall in their HFD fed counterparts, in which the hypoglycemic range was not reached (chow *ad libitum* vs. HFD *ad libitum*; Figures 5A,B). In hypoxic mice as compared to their weight matched controls, the ITT suggested worsening of insulin sensitivity: Although starting off with lower blood glucose (*p* = 0.003), hypoxia treated mice showed significantly higher glycemia at the end of the ITT (*p* = 0.008; HFD restricted vs. HFD+hypoxia; Figure 5A). The data shown in Figure 5 even somewhat underestimates the glucose lowering response of the restrictedly fed animals, because the ITT of 2 mice belonging to this group had to be discontinued, when blood glucose values fell to less than 2 mmol/l (one at 30 min, one at 45 min). All mice from the other groups completed the ITT.

### Hepatic steatosis (Figure 6)

**Figure 6:**
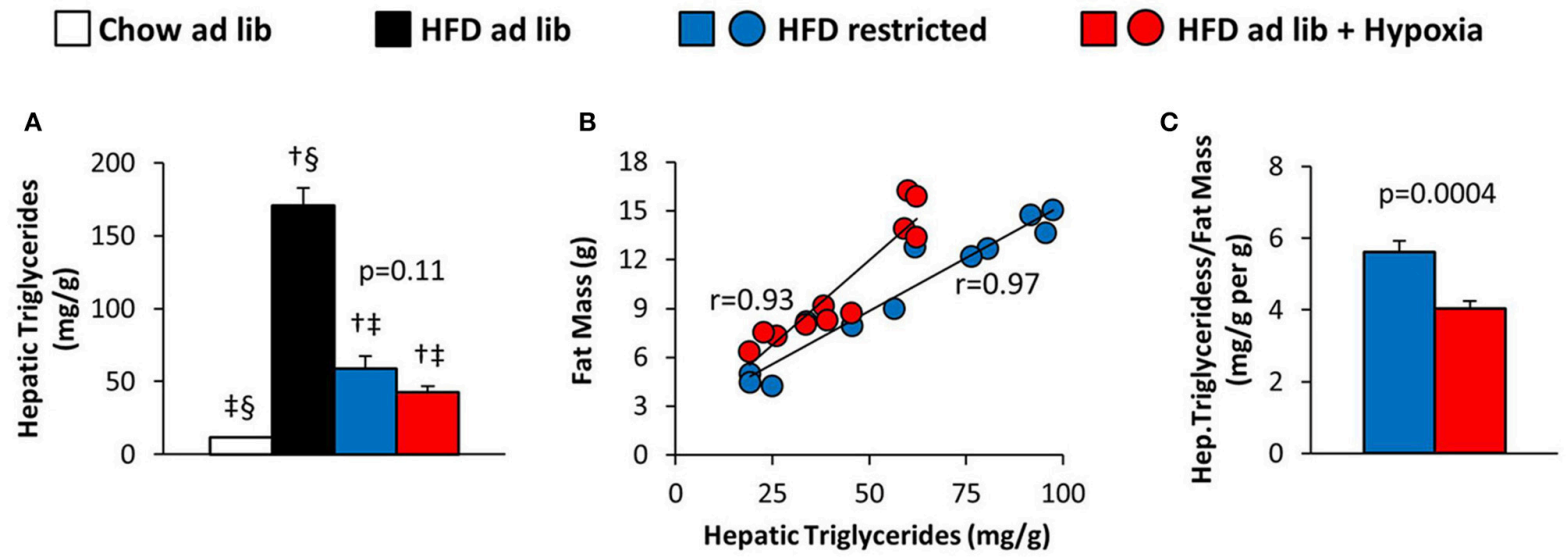
**(A)** Hepatic triglyceride content in four groups of mice after 3 months under different conditions: free access to chow diet (Chow ad lib; *n* = 8); free access to high fat diet (HFD ad lib; *n* = 8); free access to HFD under hypoxia (HDF ad lib + Hypoxia; *n* = 12); restricted access to HFD, weight-matched with the hypoxia-exposed group (HFD restricted; *n* = 12). **(B)** Intraindividual correlations of hepatic triglyceride content with total fat mass, and **(C)** hepatic triglyceride content normalized to fat mass in the hypoxia exposed and restrictedly fed groups. Means ± SEM; *p* < 0.05 by Student's *t*-test: ^†^vs. Chow ad lib, ^‡^vs. HFD ad lib, ^§^vs. HFD restricted.

Hepatic triglyceride content distinctly increased with HFD induced obesity, which was partly reversed by restricted feeding or hypoxia. However, in response to hypoxia there was a remarkable trend toward a decrease in hepatic steatosis as compared to weight-matched controls (HFD restricted vs. HFD+hypoxia; by −28%; *p* = 0.11; Figure 6A). Closer inspection revealed a strong intraindividual association between the hepatic triglyceride concentration and whole body fat mass in both, the hypoxic group and their weight-matched controls (Figure 6B; one mouse from the hypoxia treated group was excluded from this analysis due to a disproportionally low fat mass in relation to hepatic fat; *p* < 0.001 by outlier test). We then calculated the ratio of hepatic steatosis per fat mass and observed a marked and significant decrease by hypoxia (HFD restricted vs. HFD+hypoxia; *p* = 0.0004; Figure 6C).

## Discussion

Numerous experimental studies have investigated the metabolic consequences of hypoxia without reaching consistent conclusions about effects on blood glucose and glucose tolerance (7, 9, 11, 12, 15, 18, 19). So far neither of them has carefully dissected direct actions from indirect actions as may arise secondary to reduced calorie consumption and body weight. In our experiment mice were grouped to similar cohorts based on body mass and blood glucose, before they were fed HFD and continuously exposed to hypoxia for 3 months. Comparison of groups with free access to HFD confirmed that living in an oxygen deficient atmosphere causes a distinct and durable decrease in food intake. At variance to preceding studies, we applied a more stringent setup and examined not only a control group with free access to HFD, but also an additional group on normal air, in which the weight curve of the hypoxic mice was mimicked by food restriction. This was achieved by providing the same amount of HFD to controls as voluntarily consumed under hypoxia and went along with parallel changes in body composition. The same amount of food consumed resulting in almost identical weight curves does not suggest that hypoxia dependent modulation of appetite went along with specific changes in energy expenditure or food efficiency. Likewise, obesity associated increases in circulating insulin, leptin, and adiponectin were ameliorated to a similar extent by hypoxia and by restricted feeding. Taken together, this suggests that, at least in our setting, the effects of hypoxia on body weight and body composition, as well as on the circulating concentrations of several secretory molecules relevant to the regulation of fuel homeostasis were the mere consequences of a spoilt appetite.

In line with previous rodent studies (11, 12, 18, 24) prolonged hypoxia distinctly decreased blood glucose, when comparison was made to controls with free access to HFD. But at variance to body weight, body composition, insulin, leptin, and adiponectin, which seemed to be affected by hypoxia in a weight-dependent manner only, comparison of weight matched groups (HFD restricted vs. HFD+hypoxia) revealed that the improvement of glucose homeostasis went beyond of what could be attributed to blunted appetite and diminished weight gain. This implicates a direct effect of hypoxia which occurred with delay, since glucose lowering seen after the first month could be explained entirely by impaired appetite and reduced body weight, whereas the weight independent component accounted for 58 and 35% of the reductions observed after 2 and 3 months, respectively. At these time points, hypoxia had completely normalized basal glycemia to values as prevailing in untreated lean mice on a conventional chow diet.

Albeit seeming somewhat counterintuitive, the ITT suggested that the direct anti-hyperglycemic effect of hypoxia was accompanied by aggravation rather than amelioration of insulin resistance. Impairment of insulin sensitivity is in line with several preceding reports on hypoxic rodents (6, 7, 12, 14), but short treatment periods and intermittent exposure regimens have in most studies been associated with derangement of glucose and lipid homeostasis (6, 7, 9, 15, 18) rather than with decreased blood glucose and improved glucose tolerance as found in the present study. Our results thus support the possibility that glucose lowering emerges after prolonged continuous exposure only. While increased stress hormones are believed to contribute to detrimental short term effects (16, 17, 19–21), plasma corticosterone as the major stress hormone was normal after long term hypoxia in our mice. Together with the observed delay in the occurrence of a direct effect on blood glucose, this suggests that fading of an initial stress response is needed to unmask the anti-hyperglycemic potential of hypoxia.

Along with improved glucose homeostasis, hypoxia independently of body weight seemed to affect the distribution of triglycerides to liver vs. fat. Since hepatic glucose production is an important determinant of basal glycemia, it is tempting to speculate that the observed shift in the relative distribution of triglycerides from liver to fat could be causally related to hypoxia-induced lowering of blood glucose. In this regard the present study does not allow final conclusions, because it was designed only to portray the phenotype arising from hypoxia, but our observation suggests potential effects of hypoxia on lipid fluxes and calls for closer inspection in future studies. This includes that FGF-21, which tended to be elevated under hypoxia, could have a causal role in mediating the observed metabolic adaptations.

In summary, the present study is to our knowledge the first to conclusively document that life under hypoxia can reduce circulating blood glucose beyond of what is the consequence of reduced food consumption and body weight. Furthermore, we show that, under weight-neutral conditions, prolonged continuous exposure to hypoxia results in a unique metabolic phenotype characterized by lower blood glucose along with evidence for reduced rather than enhanced insulin sensitivity.

## Ethics statement

The study was carried out in accordance with the principles of good laboratory animal care and in line with effective national and international guidelines and law. The approval procedure included critical examination of all procedures and protocols by an internal expert committee at the Medical University of Vienna, as well as by another expert committee authorized by the Austrian Federal Ministry of Science, Research and Economy. The study was carried out in accordance with the recommendations from these committees and it was approved by the ministry (approvals # BMWF-66.009/0296-II/3b/2012 and BMWFW-66.009/0122-WF/II/3b/2014).

## Author contributions

SA, TS, and CF conceived and designed the experiments. SA, MH, MK, M-PW, DK, and CF performed the experiments and measurements. All authors contributed to the analysis and interpretation of results as well as to proof-reading and correction of the manuscript. CF wrote the paper.

### Conflict of interest statement

The authors declare that the research was conducted in the absence of any commercial or financial relationships that could be construed as a potential conflict of interest.
